# Encapsulation of Essential Oils via Nanoprecipitation Process: Overview, Progress, Challenges and Prospects

**DOI:** 10.3390/pharmaceutics12050431

**Published:** 2020-05-07

**Authors:** Narimane Lammari, Ouahida Louaer, Abdeslam Hassen Meniai, Abdelhamid Elaissari

**Affiliations:** 1 Univ Lyon, University Claude Bernard Lyon-1, CNRS, LAGEPP-UMR 5007, F-69622 Lyon, France; nanjbba@hotmail.com; 2 Environmental Process Engineering Laboratory, University Constantine 3, Salah Boubnider, 25000 Constantine, Algeria; wlouaer@yahoo.fr (O.L.); meniai@yahoo.fr (A.H.M.)

**Keywords:** essential oils, nanoencapsulation, polymeric nanoparticle, nanoprecipitation

## Abstract

Essential oils are of paramount importance in pharmaceutical, cosmetic, agricultural, and food areas thanks to their crucial properties. However, stability and bioactivity determine the effectiveness of essential oils. Polymeric nanoencapsulation is a well-established approach for the preservation of essential oils. It offers a plethora of benefits, including improved water solubility, effective protection against degradation, prevention of volatile components evaporation and controlled and targeted release. Among the several techniques used for the design of polymeric nanoparticles, nanoprecipitation has attracted great attention. This review focuses on the most outstanding contributions of nanotechnology in essential oils encapsulation via nanoprecipitation method. We emphasize the chemical composition of essential oils, the principle of polymeric nanoparticle preparation, the physicochemical properties of essential oils loaded nanoparticles and their current applications.

## 1. Introduction

For millennia, nature has been considered a valuable source of medicinal agents and an exciting number of modern drugs have been derived from natural sources [1]. Recently, scientists throughout the world have shifted their main focus toward herbal medicine as a form of complementary or replacement therapy [2]. In fact, the World Health Organization (WHO) estimated that between 70 and 95% of the world’s inhabitants rely mainly on herbal medicines as their primary source of medication [3]. Among the vastness of plant products, essential oils (EO) earn particular attention [4]. 

Essential oils are generally complex mixtures of volatile organic compounds biosynthesized as secondary metabolites determining the specific aroma, flavor and fragrance of plants [5,6]. Essential oils could be extracted from different plant organs by different extraction methods [7,8]. In recent decades, investigations in new technologies have led to the emergence of new innovative and more efficient extraction processes [9,10,11]. Essential oils have been used since ancient times in various cultures for medicinal and health purposes such as antibacterial, antiviral, antifungal, anticarcinogenic, antimutagenic, anti-inflammatory and antioxidant. The diverse health benefits associated to the consumption of EO or their derivatives have been extensively documented [12,13,14]. In parallel with medicinal and health purposes, the application of EO is widening to the food industry, food packaging and agriculture [15,16,17]. In each case, EO are replacing synthetic chemical products that are more toxic, or to which pests or bacteria have shown resistance [18]. Several reports highlighted the efficiency of EO over chemical preservatives in preventing the growth of pathogens and delaying food spoilage [19,20]. Moreover, they do not exhibit the harmful health risks associated with the use of synthetic pesticides. Thus, EO are today at the forefront of food and agriculture fields [21]. However, their use is always confronted by several factors including their high volatility and high risk of deterioration upon direct exposure to heat, humidity, light, or oxygen [22]. Recently, polymeric nanoparticles (NP) have been developed to encapsulate EO, shielding them with good stability, controlled delivery, enhanced bioavailability and improved efficacy [5,23,24]. Almeida et al. revealed the superior anti-herpetic activity with a controlled release of *Cymbopogon citratus* DC. EO when encapsulated by poly (lactide-co-glycolide)-NP as compared to the free oil [25]. Moreover, Choi et al. found that encapsulation of eugenol into poly-ε-caprolactone nanoparticles could improve its stability against light oxidation [26]. In another work study, the heat resistance of *Jasminum officinale* L. EO was increased after encapsulation in gelatin and arabic gum nanoparticles [27].

Among the several methods used for developing polymeric NP, the nanoprecipitation method (or solvent displacement) seems to be the most simple and reproducible [28,29]. In this review, we focus on the nanoprecipitation process to develop EO encapsulating polymeric NP, give a scope about the mechanism of nanoparticle formation, the most used raw materials, the physicochemical properties of EO loaded-NP and their application in several fields.

## 2. Essential Oils

### 2.1. Chemical Composition of Essential Oils

According to the French Agency for Normalization (AFNOR) [30], an essential oil is “the product obtained from a vegetable raw material, either by steam distillation or by mechanical processes from the epicarp of Citrus, or “dry distillation””. According to the European Pharmacopoeia [31], an essential oil is defined as an “odorous product, usually of complex composition, obtained from a botanically defined plant raw material by steam distillation, dry distillation, or a suitable mechanical process without heating”. 

Essential oils could be obtained from flowers (Citrus sinensis L., Lavandula dentata L.), leaves (Eucalyptus globulus L., Thymus vulgaris L., Mentha piperita L., Satureja hortensis L.), rhizomes (*Zingiber officinale* L., *Acorus calamus* L.), seeds (*Carum carvi* L., *Coriandrum sativum* L.), fruits (*Foeniculum vulgare* L., *Pimpinella anisum* L., *Citrus limon* L.) and woods (Cinnamomum Cassia presl., Santalum album L.) [32]. Generally, EO represent less than 5% of the vegetal dry matter. Their composition may vary with the part of the plant used as raw material, the cultivation, the soil and climatic conditions and the harvesting time [33]. Essential oils are soluble in organic solvents (alcohol, ether, and fixed oils), while insoluble in inorganic ones (water). They are volatile, liquid and colorless at room temperature; having a characteristic odor with a density less than unity except for EO extracted from Cinnamomum Cassia presl., Sassafras albidum Nutt., and *Vetiveria zizanioides* L. [32].

**Figure 1:**
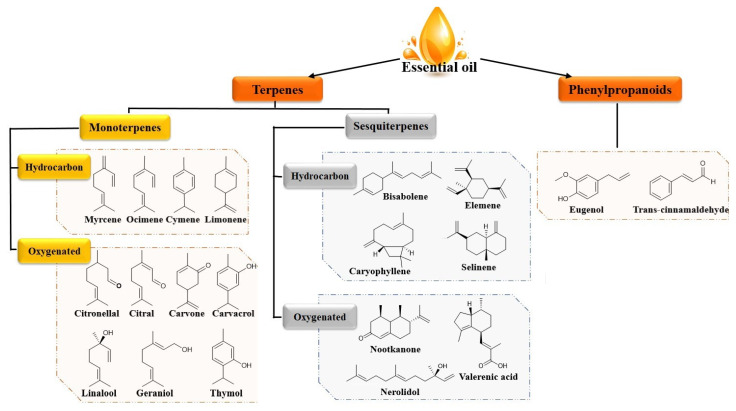
Chemical structures of some constituents of essential oils.

Essential oils are basically a complex mixture of terpenic hydrocarbons, especially monoterpenes and sesquiterpenes, and oxygenated derivatives like aldehydes (citronellal, sinensal), ketones (menthone, p-vetivone), alcohols (geraniol, α-bisabolol), phenols (thymol) and esters (γ-tepinyl acetate, cedryl acetate) [32]. Essential oils contain also non terpenic compounds known as phenylpropanoids which give a specific flavor and odor when they are present. Eugenol and cinnamaldehyde are examples of this group of constituents [6]. Figure 1 demonstrates the chemical structure of some constituents of essential oils.

### 2.2. Challenges in Rational Use of Essential Oils

Essential oils derived from different parts of aromatic plants have been extensively researched for their nutritional health benefits. Essential oils exhibit excellent antimicrobial properties and the mechanism of action has been studied in detail [34]. The main feature of EO is their hydrophobicity which allows their partition into lipids of bacterial cell membrane, leading to disrupt the structure, and make it more permeable. Several works describe even broadly known EO, like, *Syzygium aromaticum* L. [35], *Mentha piperita* L. [36], *Origanum vulgare* L. [37,38], *Cinnamomum cassia* Persl. [35], *Rosmarinus officinalis* L. [38], *Cymbopogon citratus* DC. [37] and *Thymus vulgaris* L. EO [39] as large spectrum antimicrobial agents. In 2019, Malik reported that EO obtained from *Thymus vulgaris* L., *Origanum vulgare* L., *Syzygium aromaticum* L., *Ocimum basilicum* L., *Myristica fragrans* Houtt. and *Petroselinum crispum* Mill. show remarkable antioxidant activities due to their phenolic structure which neutralize free radicals and decompose peroxides [40]. Essential oils are also used as potential anti-inflammatory agents in the treatment of arthritis, allergies and rheumatism [41,42]. Moreover, other work studies reported that EO extracted from *Melissa officinalis* L. [43], *Saussurea lappa* L. [44], *Artemisia herba-alba* Asso. [45], *Melaleuca alternifolia* Cheel. [46], and *Comptonia peregrina* L. [47] show excellent anticancer properties. Currently, the use of EO has broadened to the food packaging and agriculture fields. For instance, numerous studies have demonstrated that EO, as well as their blends, possess excellent repellent and insecticidal activities [48,49]. Furthermore, EO have shown more efficiency over chemical preservatives in preventing the growth of pathogens and delaying food spoilage [19,20].

The use of EO or their derivatives is always confronted by their volatility, chemical un-stability upon exposure to air, light, moisture and heat [22]. Hădărugă et al. reported that thermal and/or oxidative labile EO can be degraded during the processing, transportation, storage and even consumption of products containing such substances to the point that they are ineffective, or even dangerous with the formation of toxic derivatives [50]. Several examples are related to these aspects, like the degradation of safrole to carcinogenic metabolites [51], the oxidation of pinene to harmful oxidized derivatives, the diepoxidation of limonene to the carcinogenic diepoxylimonene [52], or the formation of oxygenated derivatives of linalool or caryophyllene causing allergenic and skin sensitization properties [53]. Additionally, the poor aqueous solubility of EO discourage their free use for clinical purposes [23].

Nanoncapsulation has been proposed as a novel approach to overcome the mentioned limitations [5,54]. Several nanostructured systems have been designed intending EO encapsulation as approach to enhance their bioavailability and bioefficacy as a result of high cellular uptake and controlled release delivery [5,23,24] According to the literature, polymer-based nanocarriers are extensively used for this purpose [55,56,57,58,59,60]. 

## 3. Polymeric Nanoparticle

**Figure 2:**
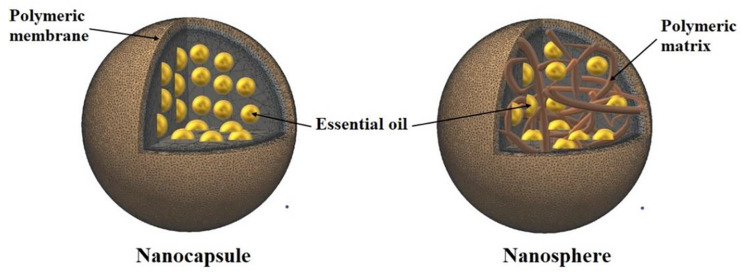
Different types of polymeric nanoparticles.

Polymeric nanoparticles are solid colloidal particles with a diameter ranging from 1 to 1000 nm. They are comprised of nanocapsules and nanospheres. Nanospheres have a monolithic-type structure (matrix) in which active pharmaceutical ingredients (API) are encapsulated within the particles or adsorbed onto their surfaces. While, Nanocapsules are the vesicular system in which the API is confined to a cavity consisting of an inner liquid core surrounded by a polymeric membrane. In this case, the API is usually dissolved in the inner core, and may also be adsorbed to the capsule surface [61] (Figure 2). With respect to EO, polymeric NP have created a tremendous interest due to their advantages over other particulate systems. For instance, they act as carriers enabling to EO a high protection against the hazardous environment, an improved stability, a reduced tissue and skin irritation and enhanced biopharmaceutical properties [23]. The use of these nanocarriers in several fields is continuously rising [62,63,64].

## 4. Nanoprecipitation Process to Encapsulate Essential Oils

### 4.1. Principle

Several preparation methods have been reported in the literature to develop polymeric NP, while the nanoprecipitation is extensively employed [55,65,66,67,68,69,70,71,72,73,74,75]. The nanoprecipitation, also called solvent displacement or interfacial deposition; was patented by Fessi et al [76]. This method involves the use of two miscible phases: an organic phase (the solvent) in which the polymer and the API are dissolved and an aqueous phase (the non-solvent). Ideally, both the polymer and the API must dissolve in the first one (the solvent) but not in the second system (the non-solvent) [29]. As a general tendency, the solvent is an organic medium, while the non-solvent is mainly water. However, it is possible to use either two organic phases or two aqueous phases as long as solubility, insolubility and miscibility conditions are satisfied [77]. 

**Figure 3:**
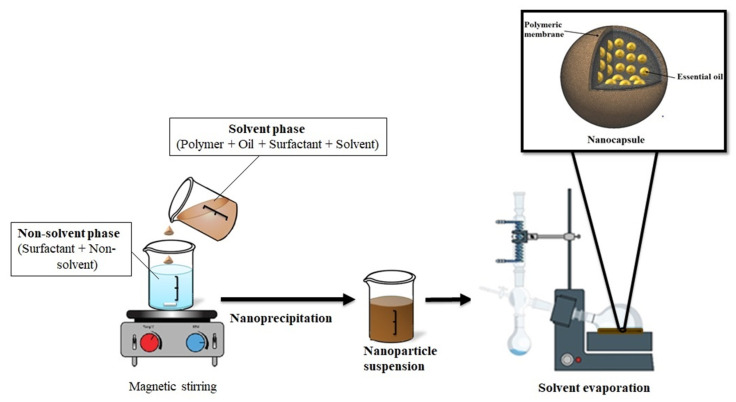
Nanoprecipitation Method.

Generally, to produce EO based-nanoparticles by nanoprecipitation method, the polymer and the EO are solubilized in an organic solvent or mixture of solvents. The organic solution is then added to water, with or without a hydrophilic surfactant under moderate magnetic stirring which causes the interfacial deposition of a polymer after displacement of the organic solvent (Figure 3). After that, the organic solvent was evaporated at ambient temperature or with a rotavapor allowing the formation of nanoparticles suspension in water [28]. The water may also be partially [78] or completely removed; rendering films [79] or powders [80].

Recently, several EO loaded-NP were developed by a modified nanoprecipitation process developed by Luque-Alcaraz et al. [74]. In this method, the aqueous phase was prepared by dissolving chitosan in acetic acid. Then, the solvent phase was added to the non-solvent phase composed of EO and methanol under moderate magnetic stirring [73,74,81,82].

### 4.2. Mechanism of Nanoparticle Formation 

**Figure 4:**
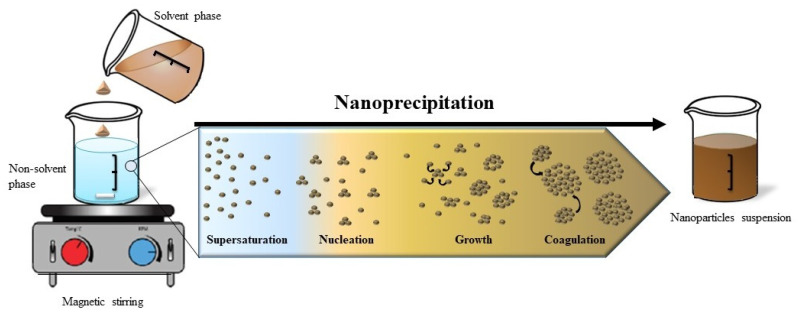
Illustration of the precipitation mechanism.

To explain the particle formation in the nanoprecipitation method, Joye and McClements pointed that this phenomenon includes four steps: supersaturation, nucleation, growth, and coagulation [83] (Figure 4). This explanation was based on the theory of Sugimoto concerning the polymer precipitation [84]. The controlling force of these phenomena is supersaturation, which is defined as the ratio of polymer concentration to its solubility in the solvent mixture. As shown in Figure 4, the addition of solvent to non-solvent decreases solvent potency to dissolve polymer, generating supersaturation, which in turn leads to polymer precipitation. After that, in order to gain thermodynamic stability, polymer particles associate and form primary nuclei; this step is known as nucleation. The formed nuclei increase in size by the association of solute molecules until it reaches a critical dimension that is stable against dissolution [83]. The fluid dynamics and mixing of phases play an important role. In fact, they influence supersaturation and owing to the rapidity of particle formation process, also determine the nucleation rate. Consequently, poor mixing produces few big nanoparticles (low nucleation rate) while good mixing conditions give birth to high nucleation rate and larger population of small particles will be formed [29]. 

When the solute concentration is reduced below the critical supersaturation concentration, nuclei growth for condensation or coagulation begins. Condensation is the addition of single molecules to the particles surface. This phase stops when solute concentration is reduced below the equilibrium saturation concentration. Coagulation is another major driving force for particle growth. It consists on the adhesion of particles to each other’s and happens when the attractive forces (Van Der Waals, hydrophobic interactions, etc.) dominate the repulsive ones (steric or electrostatic repulsion). By applying mechanical forces like stirring, homogenization, or ultrasound, the particles within the aggregate may be released. On the other hand, the nanoparticles may coalesce via aggregation process leading to the formation of stable particles. The coagulation step depends on collision frequency and efficiency. The collision frequency is the number of collisions per unit time per unit volume, and vary with the variation of particle concentration, particle size, and particle motion. Whereas, the collision efficiency represents the number of collisions which lead to coagulation, and depends on the balance of attractive and repulsive forces between the particles. To stabilize particles against coagulation, stabilizing agents could be added during the process [85].

### 4.3. Raw Materials

#### 4.3.1. The Solvent Phase

The nanoprecipitation method involves the use of an organic solvent, which is completely soluble in the external aqueous phase, inducing immediate polymer precipitation. According to the Table 1, several organic solvents could be used like, dimethyl sulfoxide [67], ethanol [75], acetic acid [72,73,74,81,82], isopropanol:acetone [68,70], tetrahydrofuran [86]; while acetone is the mostly used [55,57,65,66,71,87,88].

The polymers used could be biodegradables and biocompatibles; synthetics or naturals. Synthetic polymers like Eudragit^®^RS100 [69,75], Eudragit^®^L100-55 [68], Eudragit^®^EPO [70], poly-ε-caprolactone [65,66,89], polylactide [55] and poly(lactide-co-glycolide) [57,67] showed good results. Several natural polymers could be used to develop pNPs, among which chitosan was extensively used in encapsulating EO [72,73,82]. In other work study, cellulose acetate was used as wall forming material [71].

Hydrophobic surfactants (W/O) could be added to the nanocapsule core to hinder the particle ‘aggregation. Sorbitan esters, like span 20 [66] and phospholipids like Epikuron^®^200 [57] and lecithin [65] are commonly used.

#### 4.3.2. The Non-Solvent Phase

It could consist of mixture of surfactants, used for avoiding particle ‘aggregation; dissolved in the non-solvent. Pluronic^®^F68 [57,65], Tween 80 [66,87] and polyvinyl alcohol [68] are examples of used surfactants. While, water was the most used non-solvent [55,57,66,67,68,71,75,87,88,90]. Methanol was also used by other work studies [73,74,81,82].

### 4.4. Physicochemical Properties of Nanoparticles Produced by Nanoprecipitation

The solvent displacement technique has been extensively used to encapsulate essential oils in polymeric NP, as illustrated in Table 1. In term of particle size, size distribution and encapsulation efficiency, the nanoparticles produced by nanoprecipitation technique owned small particle size, narrow distribution with high encapsulation efficiency. For instance, Jummes et al. developed *Cymbopogon martini* Roxb. EO loaded poly-ε-caprolactone based-nanocapsules with small particle size (282.1 nm), narrow size distribution (poly dispersibility index less than 0.14) and high encapsulation efficiency (99.54%) [65]. Similarly, *Rosmarinus officinalis* L. EO was efficiently entrapped in poly-ε-caprolactone based-NP with an average size of 220 nm, zeta potential equal to −19.9 mV and an encapsulation efficiency of about 99% [66]. Furthermore, carvacrol loaded-poly (lactide-co-glycolide) based-nanocapsules exhibited a spherical shape, small particle size (209.8 nm) and regular distribution (poly dispersibility index around 0.26) [57]. 

According to the literature, several works reported the effectiveness of the nanoprecipitation over other methods in term of particle size and oil entrapment efficiency. Shakeri et al. reported that using the nanoprecipitation technique to develop carvacrol loaded-poly (3-hydroxybutyrate) nanocapsules allowed significantly improvement in oil loading (21%) and particles stability (zeta potential of −26 mV) as compared to the dialysis method [91]. Furthermore, Esfandyari-Manesh and coworkers compared the average diameter, size distribution and oil loading of poly (lactide-co-glycolide) carvone/anethole-NP prepared by either solvent displacement or emulsification solvent evaporation techniques. Results revealed that particles produced by nanoprecipitation process had smaller particle size (126 and 158 nm for carvone and anethole, respectively), narrower size distribution (poly dispersibility index about 0.08) with higher oil loading (12.32% and 14.73% for carvone and anethole, respectively). The low EO loading for the emulsification method was attributed to its loss during the evaporation phase which has taken a long time (≈3 h). Additionally, the difficulty for the mutual dispersion of the organic phase using this method results in larger particles [67]. Fraj et al. reported similar results in term of particle size, zeta potential and encapsulation efficiency of *Origanum vulgare* L. EO loaded- poly-ε-caprolactone NP prepared by two methods: nanoprecipitation and double emulsion [92]. For instance, the particle size, zeta potential and encapsulation efficiency values were 181.6 nm, −40.9 mV, 85.89% and 1759 nm, −15.7 mV, 47.5% for nanoprecipitation and double emulsion, respectively. Moreover, long-term stability study carried out for a period of 60 days at 4, 25 and 40 °C revealed that particles produced by nanoprecipitation were physically stable with high carvacrol retention; whereas, those produced by double emulsion method exhibited an increase in particle size and a decrease in carvacrol retention when treated at 25 and 40 °C [92]. 

Currently, several reports have been investigated to determine the effect of raw materials on the physicochemical properties of the NP prepared by solvent displacement method. In this context, the impact of poly-ε-caprolactone amount on the average diameter of *Rosmarinus officinalis* L. EO loaded-nanoparticles was studied. Results showed that large particles have been produced when increasing poly-ε-caprolactone quantity. Apparently, high polymer amount results in increasing the thickness of the polymeric shell and thus increasing the particles size instead of their number [66]. With regards to the effect of the type of EO, Liakos et al. encapsulated peppermint, cinnamon and lemongrass EO extracted from *Mentha piperita* L., *Cinnamomum Cassia* presl. and *Cymbopogon citratus* DC., respectively in cellulose acetate based-NP and studied the variation in the particle size. Results revealed that the particle size was in the following order: Cinnamon EO-NP < Peppermint EO-NP < Lemongrass EO-NP [71]. This finding was attributed to the chemical structure of the encapsulated EO. It seems that geranial and neral, the main components of lemongrass EO, have long carbon chains (≈10 C atoms) resulting in an increase in nanoparticle size when attached to cellulose acetate. While for cinnamon EO, its main component (cinnamaldehyde) will react with hydroxyl group of cellulose acetate creating hemiacetal bound which is responsible to combat the nanocapsules; thus, a small particle size was assessed [71]. With respect to the surface charge of NP, an increase in lemongrass EO lead to increase the zeta potential of cellulose acetate NP [71]. Authors linked the variation in zeta potential to the presence of some EO molecules onto the outer surface of the particles. In another work study, the presence of lime EO extracted from the peels of *Citrus aurantifolia* Christm. on the surface of chitosan-NP decreased the zeta potential from +61.1 to +57.0 mV after EO encapsulation [82]. This finding was attributed to the diminution of free NH_3_^+^ groups of chitosan following their interaction with lime EO [82]. In other cases, the zeta potential did not change after *Rosmarinus officinalis* L. EO encapsulation due to its presence in the core of the NP instead of being adsorbed on their surface, as previously reported [93]. By the means of the nanoprecipitation technique, polymeric nanoparticles with sufficient colloidal stability may be produced without any need for chemical surfactant. Currently, several reports highlighted the negligible effect of surfactant on the physicochemical properties of the developed NP. In this context, several EO have been efficiently entrapped in polymeric NP without adding any surfactant to stabilize the system [55,67,69,70,71,72,74,75,81,82,86,88]. In some cases, some EO played the role of surfactant-like substance facilitating the formation of NP [55,71]. As reported earlier, since peppermint, cinnamon and lemongrass EOs extracted from *Mentha piperita* L., *Cinnamomum Cassia* presl. and *Cymbopogon citratus* DC., respectively; have in their chemical structure long hydrocarbon tails and heads with functional groups, it is expected that they can act also as a surfactant for the preparation of NP [71]. Additionally; the presence of aldehydes in lemongrass oil can create aldol reactions with polylactic acid esters and thus lead to stabilize the formulation [55].

In addition to the factors mentioned above, operating conditions may affect the physicochemical properties of the NP prepared by nanoprecipitation process. In this context, Qiu and coworkers studied the effect of the complexation temperature of starch (30, 60 and 90 °C) on the colloidal properties of methone-loaded starch NP [58]. Results showed that the mean diameter of nanoparticles formed at 30, 60, and 90 °C was 112, 104 and 93 nm, respectively. Authors related the small diameter at high temperature to the slow nucleation rate which in turn led to a more ordered crystalline structure [58]. In another research work, the influence of the organic solvent elimination method on the NP properties and *Rosmarinus officinalis* L. EO loss was investigated [66]. In this study, three evaporation methods were carried out: i) Evaporation under reduced pressure at 40 °C, ii) Evaporation under reduced pressure at room temperature and iii) Evaporation under normal pressure at room temperature. Results revealed that the first method was very fast (30 min) and led to a loss of nearly 50% of the amount of the encapsulated EO due to its volatility at this temperature. In addition, the evaporation under reduced pressure at room temperature spent more time (60 min) and 50% of encapsulated EO was lost since it is evaporated at low pressure with acetone. Even though the evaporation of acetone under normal pressure at room temperature lasted longer than the first two methods (120 min), it was selected as the best method since it avoided heat and low pressure, leading to negligible loss of the EO [66]. Similar results were already described [55,67,71,93]. In parallel, for other EO the evaporation under reduced pressure at high temperature was found efficient to eliminate the organic solvent; and the developed NP showed high oil entrapment efficiency [81,82,87,90,94]. In other research work, the evaporation under low temperature and reduced pressure conditions seems efficient for acetone elimination [69]. One may conclude that the choice of the evaporation method depends on the nature of the encapsulated EO.

In vitro release of EO from particles prepared by nanoprecipitation technique generally exhibits a controlled manner. In a study carried out by Christofoli and coworkers, *Zanthoxylum rhoifolium* L. EO from poly-ε-caprolactone-NP have reached a level of maximal release (81.9%) at 72 h [87]. The release kinetic profile of *Zanthoxylum rhoifolium* L. EO showed an initial burst effect, followed by a slow release after 12 h. Similar biphasic release profile was already described [57,73,88]. As reported earlier, the initial burst release was related to the molecules that are adsorbed to the polymeric wall, while the second release phase was attributed to the EO molecules present in the core of the nanocapsules which make more time to diffuse through the polymeric wall [57]. Several factors like the types of polymeric wall and encapsulated oil were found to affect the in vitro release profile. From chitosan based-NP, more release of carvacrol was found as compared to thymol [73]. The maximum release times were 540 and 630 min for 100% release of carvacrol and thymol, respectively. This was explained by the fact that carvacrol is more hydrophilic than thymol, thus in contact with water, more release is expected for carvacrol [73]. Moreover, Popiolski and coworkers reported that *Lavandula dentata* L. EO release from polyethylene oxide-b-polylactic acid (PEO-b-PLA) copolymers based-NP depends on polylactide molecular weight [88]. For instance, the release of *Lavandula dentata* L. EO from PEO5KDa-b-PLA10KDa nanoparticles was about 40% and this percentage remained the same for 24 h of releasing process. While, for PEO5KDa-b-PLA4.5KDa nanoparticles, only 5% was released. This difference in release amount was related to the nanoparticle average size. In fact, the nanoparticles produced from PEO5KDa-b-PLA10KDa copolymer were significantly smaller than those produced from the PEO5KDa-b-PLA4.5KDa copolymer. Apparently, small nanoparticles favored the diffusion of lavender EO from the inner core to the external medium, leading to increase the amount of EO released [88].

The great challenge that hinders the feasibility of biological applications of EO in several fields is their instability in the presence of light, heat and humidity [22]. Hence, the encapsulation of EO in NP seems to be a promising approach. The stability of the NP produced by nanoprecipitation technique was widely investigated in the literature. In 2018, Badri et al. investigated the stability of *Nigella sativa* L. EO co-loaded with indomethacin in poly-ε-caprolactone -NP during one month under three different temperatures (4, 25 and 40 °C) [90]. No significant change was assessed in term of size or zeta potential for all the tested particles. Similarly, the protection provided by poly-ε-caprolactone nanospheres in terms of the stability for *Zanthoxylum rhoifolium* L. EO against photodegradation (UV-A and UV-C radiations) was investigated [87]. Results revealed that un-encapsulated EO suffered 94.33% photodegradation after 7 h of exposure to light, while the nanospheres degradation was only 44.76%. This was explained by the property of polymeric wall to protect the plant oils [87]. 

With the development of nanocarriers design strategies, scalability and reproducibility remain crucial in the choice of a suitable technique to produce plant oils loaded nanocarriers with predictable properties. With regards to the nanoprecipitation process, Ephrem and coworkers compared the colloidal properties of *Rosmarinus officinalis* L. EO loaded-nanocapsules prepared by this technique at small and large scales [66]. Results revealed that NP prepared at both scales were spherical in shape with an average diameter around 230 nm, polydispersity index less than 0.25, negative zeta potential around −20 mV with an encapsulation efficiency higher than 99% and good stability over time [66]. 

Overall, based on the literature discussed above, the extensive use of nanoprecipitation process for essential oil encapsulation purpose was related to its benefits including (i) rapidity [28], (ii) simplicity [29], (iii) good reproducibility [66], (iv) scalability [66] (v) no need for using high energy input [67], and (vi) the instantaneous formation of submicron nanoparticles, with narrow size distribution and high encapsulation efficiency [57,91,93]. 

**Table 1:** Nanoprecipitation method for encapsulating essential oils in polymeric nanocapsules.

Essential Oil	Source of Essential oil	Part of the Plant	Solvent Phase			Non Solvent Phase					Biological Properties	Application	Ref.
			Polymer	Surfactant	Solvent	Surfactant	Solvent	Size (nm)	Z. Pot (mV)	EE (%)			
Palmarosa	*Cymbopogon martini* Roxb.	Leaves	PCL	Lecithin	Acetone	Pluronic F68	Water	282.1	−27.2	99.54	Antioxidant Antimicrobial	Cosmetic	[65]
Thyme	*Thymus vulgaris* L.	Stem + leaves	Eudragit^®^L100-55	/	Acetone: Isopropanol	PVA	Water	153.9	−4.11	52.81	Antioxidant	Food	[68]
	*Thymus serpyllum* L.	Stem + leaves	Chitosan	/	Acetic acid	/	Methanol	/	/	68	Antimicrobial	Agriculture	[73]
			Chitosan	/	Acetic acid	/	Methanol	117–226	+27	/	Antimicrobial	Agriculture	[81]
	*Thymus leptobotrys* L.	Aerial part	Eudragit RS 100	/	Ethanol	/	Water	144	+80.9	/	Bacteriostatic Fungistatic	Medicine	[75]
	*Thymus satureoides* L.	Aerial part	Eudragit RS 100	/	Ethanol	/	Water	132	81.6	/	Bacteriostatic Fungistatic	Medicine	[75]
Bergamot	*Citrus bergamia* Risso.	Peels of fruit	Eudragit^®^RS100	/	Acetone	/	Water	57 to 208	39 to 74	28–84	Antimicrobial	Food	[69]
Sweet orange	*Citrus sinensis* L.	Peels of fruit	Eudragit^®^RS100	/	Acetone	/	Water	57 to 208	39 to 74	56–96	Antimicrobial	Food	[69]
Oregano	*Origanum Vulgare* L.	Leaves	PCL	Span 80	Acetone	Tween 80	Water	181.6	−40.7	85.9	Antimicrobial	Textile	[95]
Rosemary	*Rosmarinus officinalis* L.	Aerial parts	Eudragit^®^EPO	/	Acetone: Isopropanol	/	Water	200	/	59	Antioxidant	Cosmetic	[70]
		Leaves	PCL	Span 20	Acetone	Tween 80	Water	145	−11	78.2	Insecticide	Agriculture	[93]
			PCL	Span 20	Acetone	Tween 80	Water	220	−19.9	99	Antioxidant Analgesic Antimicrobial	Medicine	[66]
Lavender	*Lavandula dentata* L.	Aerial parts	Eudragit^®^EPO	/	Acetone: isopropanol	/	Water	200	/	41	Antioxidant	Cosmetic	[70]
			PEO-B-PLA	/	Acetone	/	Water	10–75	/	70–75	Antimicrobial Sedative	Textile	[88]
Nigella	*Nigella sativa L.*	Seeds	PCL	/	Acetone	PVA Tween 80	Water	230–260	−30 to −20	/	Anti-inflammatory	Cosmetic	[90,96]
Peppermint	*Mentha piperita* L.	Aerial parts	Cellulose acetate	/	Acetone	/	Water	180	−38	/	Antimicrobial	Medicine	[71]
Cinnamon	*Cinnamomum Cassia* presl.	Bark	Cellulose acetate	/	Acetone	/	Water	150	−40	/	Antimicrobial	Medicine	[71]
Lemongrass	*Cymbopogon citratus* DC.	Leaves	Cellulose acetate	/	Acetone	/	Water	200	−36	/	Antimicrobial	Medicine	[71]
		Leaves	PLA	/	Acetone	/	Water	300	−6	/	Antimicrobial	Medicine	[55]
Pepper tree	*Shinus mole* L.	Leaves	Chitosan	/	Acetic acid	/	Methanol	355.3	/	/	Antifungal	Food	[72]
		Leaves	Chitosan	/	Acetic acid	/	Methanol	754	+9.1	/	Antifungal	Agriculture	[74]
Lime	*Citrus aurantiifolia* Christm.	Peels of fruit	Chitosan	/	Acetic acid	/	Methanol	/	+10	/	Antimicrobial	Food	[82]
		Peels of fruit	Chitosan	/	Acetic acid	/	Methanol	250	+10	/	Antimicrobial	Agriculture	[81]
Geraniol	/	/	PluronicF-127	/	THF	/	Water	26–412	/	/	Antimicrobial	Food	[86]
			PCL	Lecithin	Acetone	Pluronic F68	Water	289.3	−26.6	99.88	Antioxidant Antimicrobial	Cosmetic	[65]
*Zanthoxylum rhoifolium*	*Zanthoxylum rhoifolium* L.	Leaves	PCL	Span 60	Acetone	Tween 80	Water	˂500	−20	96	Pesticide	Agriculture	[87]
*Pelargonium graveolens*	*Pelargonium graveolens* L’Hér.	Aerial part	Eudragit RS 100	/	Ethanol	/	Water	113	+80.6	/	Bacteriostatic Fungistatic	Medicine	[75]
*Eugenia Caryophyllata*	*Eugenia Caryophyllata* C.	Buds	Eudragit RS 100	/	Ethanol	/	Water	131	+80.7	/	Bacteriostatic Fungistatic	Medicine	[75]
Carvone	/	/	PLGA	/	DMS	/	Water	126	/	61	Antimicrobial	Food	[67]
Anethole	/	/	PLGA	/	DMS	/	Water	158	/	87	Antimicrobial	Food	[67]
Thymol	/	/	Ethyl cellulose Methyl cellulose	/	Ethanol	/	Water	420	/	77	Antimicrobial	Cosmetic	[97]
Carvacrol	/	/	PLGA	Epikuron 200	acetone	Pluronic F68	Water	209	−19	26	Antimicrobial	Medicine	[57]

PCL, Poly-ε-caprolactone; PVA, Polyvinyl alcohol; PEO-b-PLA, Polyethylene oxide-b-Polylactic acid) copolymers; PLA, Polylactide; THF, Tetrahydrofuran; PLGA, Poly(lactide-co-glycolide); DMS, Dimethyl sulfate.

## 5. Applications

Essential oils have conquered space in several fields due to their pertinent properties. Currently, the biological effects of EO have been widely documented [32,98]. The Table 1 summarizes some work studies concerning the application of EO-loaded polymeric NP prepared by nanoprecipitation process.

### 5.1. Agriculture Field

During the last few decades, environmental risks to humans, flora, and fauna and the development of resistance in species of pathogenic microorganisms have increased significantly due to the indiscriminate use of synthetic agrochemicals such as pesticides, herbicides and insecticides. Today, the use of natural compounds especially EO in the agriculture field has brought to the forefront [87,99,100]. *Aspergillus flavus* and *Aspergillus parasiticus* are widely involved in food spoilage producing secondary metabolites or mycotoxins that are carcinogenic and cause fatal diseases in both animals and humans. Recently, Luque-Alcaraz et al. investigated the inhibitory effect of *Schinus molle* L. EO loaded chitosan NP against the filamentous fungi *Aspergillus parasiticus* involved in spoilage of fruits, vegetables or other substrates rich in carbon sources [74]. Apparently, chitosan is a biologically compatible polymer having a significant effect in the control of phytopathogenic fungi, Gram positive and Gram-negative bacteria [101,102,103]. The developed NP exhibited a large decrease (40–50%) in *Aspergillus parasiticus* viability. Similarly, Sotelo-Boyas et al. reported that the inclusion of thyme EO extracted from the stem and leaves of *Thymus serpyllum* L. in chitosan NP is a feasible alternative to obtain antibacterial nanoparticles, where the activity that each compound presents individually is strengthened [73]. The highest inhibitory activity was observed against *Staphylococcus aureus* (Inhibition halo = 4.3 cm) for 40 µL of minimum inhibitory volume [73]. Sotelo-Boyas et al. in another work study, developed chitosan nanoparticles embedding two types of EO: Thyme and lime EO, extracted from *Thymus serpyllum* L. and *Citrus aurantifolia* Christm., respectively; to eradicate *Pectobacterium carotovorum*, plant phytopathogenic bacteria, involved in the decay of fresh fruits and vegetables like potatoes, carrots, radishes, onions, cucumbers, squash, eggplant, peppers, cabbage and tomato [81]. Thyme EO based-NP showed the highest inhibitory effect on the growth of *Pectobacterium carotovorum* than free chitosan NP and lime EO based-NP. For chitosan free nanoparticles, the number of colonies forming units (CFU) could not be determined (countless colonies). While for chitosan-lime EO- and chitosan-thyme EO- based NP, CFU were found 450 and 240 respectively. The inhibition halo diameter of free chitosan-NP, chitosan-lime EO-NP and chitosan- thyme EO-NP were found 15.0 ± 0.2, 13.0 ± 0.1 and 24.0 ± 0.1 mm, respectively [81]. Furthermore, Christofoli et al. developed a promising pesticide based on *Zanthoxylum rhoifolium* L. EO loaded in poly-ε-caprolactone nanospheres and investigated *in vivo* activity against *Bemisia tabaci* populations using tomato as host plant [87]. The biological assays revealed that the developed NP significantly reduce the number of eggs and nymphs in a dose-dependent manner. Indeed, the nymphs number reduction was about 83, 89, 92, and 98% for the concentrations of 0.5, 1, 2 and 5%, respectively. While, the egg-laying was about 71, 77, 83 and 96% for the same concentrations [87]. In another work study, Khoobdel et al. reported that the encapsulation of *Rosmarinus officinalis* L. EO in poly-ε-caprolactone NP enhances its insecticidal activity against the red flour beetle, *Tribolium castaneum* [93]. One may conclude that the biological effect of EO was enhanced upon nanoencapsulation in polymeric NP due to the increased surface area of the nanoparticles and the controlled release of EO.

### 5.2. Food Field

The use of synthetic food preservatives is the most common method of postharvest disease control; however, due to the high consumer awareness to the use of synthetic additives, formulations based on low toxicity and more environmentally friendly compounds are more desirable. In light of that, natural extracts like EO or their main components, which are considered as Generally Recognized as Safe (GRAS) are effective alternatives to synthetic products. Currently, several works have evidenced the application of EO as antimicrobial and antioxidant in food packaging [104]. In 2017, Sotelo-Boyás and coworkers developed *Citrus aurantifolia* Christm. EO loaded-NP by nanoprecipitation technique and tested their antibacterial activity against four food-borne bacteria: *Staphylococcus aureus, Listeria monocytogenes, Shigella dysenteriae* and *Escherichia coli* [82]. The highest inhibition was found against *Shigella dysenteriae*, with an inhibition halo diameter of 3.5 cm for 40 mL of minimum inhibitory volume [82]. Furthermore, the encapsulation of anethole and carvone in poly (lactide-co-glycolide)-NP improved their antimicrobial activity against *Salmonella typhi, Staphylococcus aureus* and *Enterococcus coli* with minimum inhibitory concentration (MIC) ranging from 182 to 374 mg/mL [67]. The enhancement in antimicrobial activity was explained by the sustained release, the improved hydrophilicity, and the better penetration resulted from small size [67]. Similarly, the antioxidant and antimicrobial activities of several EO including menthone, oregano, cinnamon, lavender, and citral were improved upon encapsulation in starch nanoparticles, as previously reported [58].

Other examples of foodborne microbial pathogens which continue to impose significant health burden even in developed countries are *Salmonella enterica* and the Shiga toxin-producing *Escherichia coli* (STEC), including *Escherichia coli* O157:H7. This pathogen can be transmitted to consumers by a variety of food vehicles such as poultry, meat and fresh and dairy products, causing harmful diseases. In 2016, Yegin et al. investigated the inhibition of *Salmonella enterica* and *Escherichia coli* O157:H7 *in vitro* on spinach surfaces by geraniol loaded-pluronic F127 nanoparticles [86]. For both *Salmonella enterica* and *Escherichia coli* O157:H7, a decrease in MIC of geraniol following nano-encapsulation was reported. For *Salmonella enterica*, the MIC values were 0.25 and 0.70 wt.% with encapsulated and un-encapsulated geraniol, respectively; while for *Escherichia coli* O157:H7, the MIC values were 0.2 and 0.4 wt.%. The type of NP application onto spinach inoculated with pathogens was investigated and results showed that the immersion was more effective than the spraying technique due to the high contact between pathogen cells attached to spinach surfaces and EO loaded-NP. In this study, more experiments were carried out to investigate the NP absorption and interaction with bacterial membrane lipids. In this context, confocal microscopy was used to characterize the interaction of fluorescent geraniol NP with *Escherichia coli* O157:H7 cells. Results showed that un-encapsulated Nile Red, used as fluorescent agent, was not absorbed into cell membranes and no fluorescent signal from cells was detected. While, nanoparticles co-encapsulated geraniol and Nile Red were taken up into the internal compartments of *Escherichia coli* O157:H7 cells as fluorescence was detected from these cells. This finding was attributed to the fact that encapsulation of EO enhances their bio-availability and transport to targeted cells [86]. 

In another work study, Chavez-Magdaleno and coworkers developed *Schinus molle* L. EO-loaded chitosan nanoparticles to eradicate *Colletotrichum gloeosporioides,* a phytopathogen fungus responsible for the anthracnose of avocado (i.e. a disease that causes loss close to 20% of the annual production) [72]. In this study, a synergic effect between chitosan and *Schinus molle* L. EO was shown; and the developed particles demonstrated a high inhibitory effect upon the in vitro viability of *Colletotrichum gloeosporioides* and low mutagenicity and toxicity. Additionally, authors reported that the inhibitory effect increased proportionally to the used concentration. At a concentration of 0.160 mg/mL, the developed particles exhibited a greater inhibitory effect on radial growth, spore germination and viability of the spores [72]. Besides chitosan, another positively charged polymer (i.e. Eudragit^®^RS100) was found to exhibit an antibacterial activity. In 2019, Froiio et al. pointed out a synergetic effect between Eudragit^®^RS100 and sweet orange EO, extracted from the peels of *Citrus sinensis* L., to preserve the fresh orange juice against foodborne bacteria: *Escherichia coli* [69]. Chavez-Magdaleno et al. related this effect to the electrostatic interactions between positive charge of the polymer and the negative charge of the membrane’s phospholipids [72].

Currently, several reports carried out in situ assays by applying coating formulations containing EO-loaded nanosystems on fresh fruits to investigate their shelf life and to evaluate any damage caused by microorganisms. In this context, Piña-Barrera et al. proposed a promising food packaging system for the preservation of grape against foodborne bacteria basing on *Thymus vulagris* L. EO loaded-Eudragit L 100-55 nanoparticle [68]. The antioxidant activity and the effect of the loaded NP on postharvest quality and shelf life of grapes (*Vitis Vinifera* L.) were carried out. The 2,2-diphenyl-1-picrylhydrazyl (DPPH) assay revealed that the radical scavenging activity was higher for encapsulated EO (73.50 ± 0.76%) as compared to that of un-encapsulated oil (59.62 ± 1.77%). Additionally, the shelf life study demonstrated that grapes treated with EO-loaded-NP maintained their characteristics of color, firmness, titratable acidity, and total soluble solid content for longer time than those without any treatment [68]. Furthermore, control grapes and groups of grapes with several coating systems (i.e. un-encapsulated EO, free-NP and EO loaded-NP) were stored for 6 months at 4 °C in order to evaluate any macroscopic damage caused by microorganisms. Results showed that all groups of grapes showed damage caused by microorganisms except of grapes treated with *Thymus vulgaris* L. EO loaded nanosystem [68]. In another research work, the efficacy of coating formulations containing *Thymus vulgaris* L. EO loaded chitosan-NP at different concentrations for controlling *Colletotrichum gloeosporioides* in vitro and in situ was investigated [94]. Results revealed a concentration-dependent effect of oil on mycelial growth and spore germination. In situ analysis showed that the disease incidence for the uncoated avocado was 84.6% which was higher than the value obtained for the coated avocado (54.0%) at the end of the storage period. Approximately, for uncoated avocado almost 50% of the fruit surface was rotten compared with coated fruit which showed less than 25% of disease symptoms. Results showed also that incorporation of *Thymus vulgaris* L. EO loaded chitosan-NP did not affect fruit quality since weight loss, dry matter content, total soluble solids and acidity were similar to that of the control fruit [94].

### 5.3. Medicinal Field

The ability to form biofilms contributes significantly to the pathogenesis of many microbial diseases [105,106] and medical device-related infections [107]. In parallel, the indiscriminate use of antibiotics has resulted in the emergence of multi-drug-resistant bacterial pathogens [108]. Therefore, growing concern about the management of bacterial infection is propelling the urgent replacement of existing antibiotics. Essential oils seem to be efficient to overcome such problems [109]. Carvacrol, the main component of several EO, has gained considerable interest due to its wide spectrum of antimicrobial activity and his ability in inhibiting the growth of preformed biofilms and interfering with biofilm formation. It has been encapsulated in poly (lactide-co-glycolide) nanocapsules using the solvent displacement technique [57]. The produced NP showed a considerable reduction in the elasticity and mechanical stability of preformed biofilms of *Staphylococcus epidermidis* [57]. Furthermore, Liakos et al. pointed out the efficacy of polylactide nanocapsules containing *Cymbopogon citratus* DC. EO to reduce the ability of *Escherichia coli* and *Candida albicans* to develop biofilms in a dose and strain dependent manner as compared to the un-encapsulated EO [55]. In addition, MTT assay carried out on the developed particles demonstrated their biocompatibility since the human amniotic fluid stem cells presented a normal metabolism and growth [55]. Afterwards, in 2018, the same research team investigated the cytotoxicity and antibacterial activity of cellulose acetate NP containing either *Mentha piperita* L., *Cinnamomum Cassia* presl. or *Cymbopogon citratus* DC. EO and results revealed no significant cytotoxicity on the normal growth and the development of cultured diploid human cells [71]. Additionally, all the NP presented a high antimicrobial effect against all the tested microbial strains (*Staphylococcus aureus, Pseudomonas aeruginosa, Escherichia coli* and *Candida albicans*).

### 5.4. Cosmetic Field

Nowadays, EO are remarkably incorporated in cosmetic products due to their ability to enhance the product’ properties and preservation, and to give a marketing image to the final product. Several work studies have been elaborated to incorporate EO in cosmetics and personal care products. Recently, Jummes et al. developed *Cymbopogon Martinii* Roxb. EO loaded-NP as an alternative to synthetic antioxidants for cosmetic application [65]. The developed particles showed high antioxidant activity against DPPH free radical and an improved antibacterial activity against *Staphylococcus aureus* and *Escherichia coli* as compared to free EO emulsion [65]. Furthermore, Badri et al. co-encapsulated *Nigella sativa* L. EO to indomethacin in order to boost the anti-inflammatory and analgesic effects of indomethacin [96]. Skin penetration on the ex vivo human skin model and in vivo study on mice were carried out. Confocal laser scanning microscopy images revealed that the developed particles showed penetrability across stratum corneum to dermis layer. Additionally, the co-encapsulated nanoparticles demonstrated highest anti-inflammatory effect compared to indomethacin particles; and the inflamed ear continued to show reduction in thickness over 8 hours of observation. This finding confirmed the synergistic and prolonged effect contributed by *Nigella sativa* L. EO [96]. In another work study, lotion, gel and cream formulations containing thymol loaded spheres were found as an effective preservative, as good as the conventionally used preservative (methylparaben), even when used at 12–52-fold lower concentrations [97]. Although the MIC and minimal bactericidal concentration (MBC) values of thymol loaded nanospheres against *Escherichia coli, Pseudomonas aeruginosa* and *Staphylococcus aureus* were in the same range as that of the unencapsulated thymol, encapsulated thymol formulation was selected as the optimal formulation since it provided an antibacterial activity for a long period. For instance, cosmetic lotion formulations containing encapsulated thymol provided total suppression of viable bacteria growth over the three months test period, while free thymol showed effective suppression for only 2–4 weeks [97]. 

### 5.5. Textiles

During recent decades, the use of EO has emerged to the textile domain and a considerable amount of research has been conducted concerning the benefits of aromatherapic textiles [110]. *Lavandula dentata* L. oil, widely used for its sedative effect, was efficiently entrapped in NP prepared from the diblock copolymer (PEO-b-PLA) for application as antibacterial agent in textiles components used in the footwear industry [88]. Various parameters have to be taken into account for the functionalization of textiles, among which the affinity between the active agent and the textile is to be considered during the application process. In light of that, Fraj et al. formulated oregano EO extracted from the leaves of *Origanum Vulgare* L., loaded poly-ε-caprolactone NP to functionalize medical devices for antibacterial activity [95]. In this study, the impregnation of the NP was performed in either synthetic polyamide or natural cotton and the amount of carvacrol, the main component of oregano EO, was quantified at the end of the padding treatment. Results revealed that cotton fabric absorbed less carvacrol than polyamide. This finding was attributed to the polyamide-poly-ε-caprolactone interaction which helps to achieve the adhesiveness of particles through textiles. It seems that the interaction was due to the hydrogen bond between the C=O present in poly-ε-caprolactone receptor and the H donor polyamide from the NH group [95].

On the whole, the polymeric NP have created a tremendous interest regarding the encapsulation of EO due to their inherent features including, (1) nanometric size which enhances cellular uptake [67,86], (2) high encapsulation efficiency [66,93], (3) high stability as compared to simple essential oil solutions, (4) protection by the polymeric wall from environmental factors (e.g. light and temperature) [87,90], and (5) bioavailability enhancement [68,111]. The main disadvantage of polymeric nanoparticles is that some traces of toxic organic solvent may remain after evaporation. However, to our knowledge no reports have been investigated regarding the toxicity of EO-loaded NP nor the quantification of the solvent traces. In recent years, several methods have been developed to produce the polymeric nanoparticles with high purity and without any trace of organic solvent [112]. In addition, when using natural polymers, the high degree of variability, the complexity and the difficulty of extraction process are the main factors that limit the use polymeric NP [113].

## 6. Conclusions 

Currently, growing concern for essential oils with the same efficiency or even more than chemical synthesized drugs has prompted scientists to focalize most of their efforts on developing new approaches to preserve the stability, bioactivity and bioavailability of these bioactive agents. The occurrence of oil nanoencapsulation has been noticed as efficient approach to resolve such restrictions. In this bibliographic paper, we emphasize an overview, recent advances, challenges and applications of essential oils loaded polymeric nanoparticles prepared via nanoprecipitation process. 

According to the literature discussed above, the nanoprecipitation technique represents an easier, less energy consuming, more reproducible, as well as a widely valid method for the encapsulation of essential oils when compared to other preparation methods. For instance, it provides most suitable nanoparticles in term of size and encapsulation efficiency. Operating conditions management and raw materials selection are key points to obtain formulations bearing suitable characteristics for the in vitro and in vivo applications. Important activities, like antimicrobial, antioxidant, antifungal, anti-inflammatory, pesticidal and insecticidal were enhanced upon the encapsulation of essential oils in polymeric nanoparticles.

Although several advances have been reported in the literature, more in vivo studies are needed to provide reliable results. Additionally, toxic effects, accumulation in biological systems and removal mechanisms must be established. Scale-up the nanoprecipitation in industries constitute another important aspect to be taken into consideration due to the fact that using polymeric nanoparticles for the delivery of essential oils is one of the newest approaches in the pharmaceutical technology.
